# Quercetin modulates signaling pathways and induces apoptosis in cervical cancer cells

**DOI:** 10.1042/BSR20190720

**Published:** 2019-08-13

**Authors:** Madhumitha Kedhari Sundaram, Ritu Raina, Nazia Afroze, Khuloud Bajbouj, Mawieh Hamad, Shafiul Haque, Arif Hussain

**Affiliations:** 1 School of Life Sciences, Manipal Academy of Higher Education, P.O. Box 345050, Dubai, United Arab Emirates; 2 College of Medicine, University of Sharjah, Sharjah, United Arab Emirates; 3 Department of Medical Laboratory Sciences and Sharjah Institute for Medical Research, University of Sharjah, Sharjah, United Arab Emirates; 4 Research and Scientific Studies Unit, College of Nursing and Allied Health Sciences, Jazan University, Jazan-45142, Saudi Arabia

**Keywords:** apoptosis, cervical cancer, chemoprevention, extrinsic pathway, Quercetin

## Abstract

Cancer cells have the unique ability to overcome natural defense mechanisms, undergo unchecked proliferation and evade apoptosis. While chemotherapeutic drugs address this, they are plagued by a long list of side effects and have a poor success rate. This has spurred researchers to identify safer bioactive compounds that possess chemopreventive and therapeutic properties. A wide range of experimental as well as epidemiological data encourage the use of dietary agents to impede or delay different stages of cancer. In the present study, we have examined the anti-ancer property of ubiquitous phytochemical quercetin by using cell viability assay, flow cytometry, nuclear morphology, colony formation, scratch wound assay, DNA fragmentation and comet assay. Further, qPCR analysis of various genes involved in apoptosis, cell cycle regulation, metastasis and different signal transduction pathways was performed. Proteome profiler was used to quantitate the expression of several of these proteins. We find that quercetin decreases cell viability, reduces colony formation, promotes G_2_-M cell cycle arrest, induces DNA damage and encourages apoptosis. Quercetin induces apoptosis via activating both apoptotic pathways with a stronger effect of the extrinsic pathway relying on the combined power of TRAIL, FASL and TNF with up-regulation of caspases and pro-apoptotic genes. Quercetin could inhibit anti-apoptotic proteins by docking studies. Further, quercetin blocks PI3K, MAPK and WNT pathways. Anticancer effect of quercetin observed in cell-based assays were corroborated by molecular biology studies and yielded valuable mechanistic information. Quercetin appears to be a promising candidate with chemopreventive and chemotherapeutic potential and warrants further research.

## Introduction

Cancer is one of the foremost causes of mortality across the world. Conventional cancer therapies lead to severe side effects, resulting in poor quality of life for the patient [1]. Therefore, there is a gradual shift toward a more targeted mechanism-based chemopreventive approach in lieu of conventional cytotoxic chemotherapeutics. Extensive epidemiological evidences suggest that a diet of fruit and vegetables can prevent a range of human cancers and are associated with a decreased risk of cancer-related mortality [2–7].

Plant polyphenols are structural group of naturally occurring organic chemicals distinguished by the existence of multiple phenolic functional units present in commonly consumed foods. Dietary polyphenols exhibit anti-inflammatory, immunomodulatory, antioxidant and pro-apoptotic properties and modulate cell signaling pathways that effectively suppress various stages of carcinogenesis [8–11]. Earlier *in vitro* studies demonstrate anticancer effect of phytochemicals derived from fruits and vegetables like genistein, EGCG, capsaicin, curcumin, sulforaphane, 6-gingerol and eugenol [12–17]. The modulation of cell signaling pathways, activation of cell death signals and induction of apoptosis in precancerous or malignant cells make phytochemicals a promising strategy in the management of malignancies [18–22].

Quercetin, a flavonoid (a subclass of polyphenolic compounds) is ubiquitously available in several vegetables and fruits. This compound has antioxidant, prooxidant, antivirus, anti-allergic and analgesic properties along with a variety of pharmacological effects [23]. Previous *in vivo* and *in vitro* experiments have demonstrated that quercetin impedes the growth of several tumors including breast, colon, ovary and stomach by inhibiting the cell cycle and cell signaling pathways (PI3K and MAPK pathways), regulating growth factors and apoptosis induction [24,25]. The prevention of colon and lung carcinogenesis by diet-derived quercetin has been demonstrated in the recent past [26,27].

The present study investigates the anti-proliferative and anti-apoptotic potential of quercetin on HeLa cells. Although, anti-proliferative potential of quercetin is known, there is no conclusive evidence available regarding its mechanistic action. In the present study, we have undertaken a comprehensive analysis of quercetin-induced apoptosis in cervical cancer cells and its effect on genes involved in apoptosis and tumorigenesis.

## Materials and methods

### Cell line and cell culture

Human cervical carcinoma HeLa cells were gifted by Dr. Tahir A. Rizvi, UAE University, Al-Ain, UAE. The cell line was maintained in Dulbecco’s modified Eagle’s medium (Sigma, St. Louis, MO) and supplemented with 10% fetal bovine serum (Sigma) and 100X Pen-strep (Sigma) in a humidified atmosphere of 5% CO_2_ in air at 37°C.

### Preparation of quercetin

Quercetin (Sigma, U.S.A.) was prepared in 66.17 mM stock using DMSO and stored at −20°C. The working concentration of 1 mM quercetin was made in a complete medium. A range of 1–150 µM quercetin was tested in MTT assay followed by utilization of sublethal doses of 25 and 50 µM quercetin for all the assays.

### Viability assay of HeLa cells and lymphocytes

Approximately 10000 HeLa cells/well were plated in 96-well plate and incubated for 24 h. After attachment, the cells were treated with different concentrations of quercetin ranging from 1 to 150 µM for 24 and 48 h. Similarly, cells were treated with vehicle control using DMSO. Morphological changes in HeLa cells were recorded using an inverted microscope (Labomed, U.S.A.). Following the treatment, MTT (Sigma–Aldrich) at final concentration of 0.5 mg/ml was added and incubated at 37°C for 2 h. The formazan crystals were solubilized with 100 µl of DMSO with 20-min incubation at 37°C (Sigma–Aldrich). Absorbance Microplate Reader (BioTek, U.S.A) was used to record the absorbance at 570 nm and calculate the viability of the cells. The experiments were repeated thrice and expressed as an average. The cell viability was calculated following the below-mentioned formula:

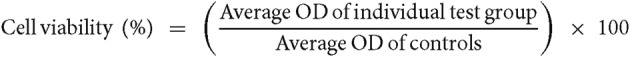


Lymphocytes were isolated from fresh blood using HiSep Media (HiMedia, India) following the manufacturer’s instructions. They were then resuspended in RPMI media and plated in 96-well microplates at approximately 10,000 cells/well and treated with quercetin as stated above. MTT assay was performed after 24 h exposure.

### Colony formation assay

Approximately 25 x 10^4^ cells were plated in six-well plates and treated the following day with 25 and 50 μM (24 and 48 h) quercetin. The cells were harvested post treatment, counted and plated at approximately 500 cells/well. After 2 weeks, the cells were fixed in 100% methanol, stained with 0.5% Crystal Violet and colonies were counted [28,29].

### Nuclear morphology analysis with propidium iodide staining

Nuclear morphology analysis using propidium iodide (PI) stain was employed to analyze whether quercetin enables apoptotic cell death in HeLa cells. Briefly, the cells (approximately 25 × 10^4^ cells/ml) were seeded on glass coverslips and left overnight to attach in a complete medium at 37°C, followed by the treatment with 25 and 50 μM quercetin (24 and 48 h). After the treatment, the cells were fixed in a mixture of acetone:methanol (1:1) at −20°C for 10 min and washed with PBS (pH 7.4) twice, and further stained with PI (10 μg/ml) for 30 s in dark at room temperature. The coverslips were then rinsed with PBS and mounted on a slide and observed at 515 nm under the Progress Fluorescent Microscope (Olympus, U.S.A.).

### Cell cycle analysis with PI staining using flow cytometry

To determine the effect of quercetin on the cell cycle, approximately 2 × 10^6^ cells were treated with 25 and 50 μM quercetin (24 and 48 h). The treated cells were fixed with 70% ethanol and incubated at −20°C. The fixed cells were washed with PBS, stained with PI (10 mg/ml PI; 0.5% Tween-20; 0.1% RNase in 0.01 M phosphate buffered saline) and processed using fluorescence-activated cell sorter (FACS; BD flow cytometer). The data were analyzed using FlowJo® software program. Untreated cells were used as control.

### DNA ladder assay

DNA fragmentation kit (Abcam, U.S.A.) was used to extract the nucleosomal fraction and to analyze the fragmentation of DNA. Briefly, approximately 1 × 10^6^ cells were plated and incubated at 37°C. The cells were treated with 25 and 50 μM quercetin for 24 h and harvested. The untreated cells were used as control. Nucleasomal DNA was extracted using the manufacturer’s protocol and electrophoresed at 80 V for 1 h on 1.2% agarose gel alongside a 100-bp DNA ladder. The samples were visualized in a gel documentation system and images were recorded.

### Single cell gel electrophoresis assay

The single cell gel electrophoresis assay or comet assay was used to detect DNA damage following quercetin treatment if any. Approximately 25 × 10^4^ cells were plated in six-well plates and treated with 25 and 50 μM quercetin (24 and 48 h). The cells were harvested after the treatment and Alkaline CometAssay® (Trevigen, U.S.A.) was performed according to the manufacturer’s protocol to detect single- and double-stranded breaks. The samples were stained with 20 µg/ml PI in PBS and then visualized using fluorescent microscope. The images were scored using the OpenComet plugin with Image Lab (www.rsbweb.nih.gov/ij/) [30]. The samples were scored on the basis of their tail length.

### Caspase 3 activity assay

Sigma’s Caspase 3 Colorimetric Assay Kit was used to detect caspase 3 activity in treated and untreated cell lysates. Approximately 1 × 10^6^ cells were plated and incubated at 37°C. The cells were treated with 25 and 50 μM quercetin (24 and 48 h) and then harvested. Untreated cells were used as control. The cells were lysed, and the assay was set up according to the manufacturer’s protocol. After overnight incubation, the plate was read at 562 nm. OD_562_ readings corresponded to caspase 3 activity and fold change with respect to the control was calculated and expressed as a graph.

### Scratch wound assay

The effect of various dosages of quercetin on tumor cell migration was examined by performing the cell migration assay as described previously [31]. The cells were seeded in six-well plates at a density of approximately 5 × 10^6^ cells per well and cultured until completely confluent. A yellow tip was used to score a constant diameter wound or cell free line. The cells were treated with 25 and 50 μM quercetin. The untreated cells were used as control. The migration of the cells across the cell free line was monitored microscopically at 0, 24, 48 and 72 h and images were obtained every 24 h. Monitoring was continued until the cell free line in the control wells reached complete closure. The wound width was measured and the percentage of wound closure was calculated and represented as a graph.

### Expression analysis of genes involved in apoptosis, tumorigenesis and cancer-related pathways using qPCR

Total RNA isolation was carried out by using Gen Elute Mammalian Genomic Total RNA Kit (Sigma) from untreated and quercetin-treated HeLa cells (25 and 50 µM for 48 h) and cDNA was synthesized (ABI RT-PCR Kit). The synthesized cDNA was then used as a template for TaqMan® Human Apoptosis Array (Thermo Fisher), which has a range of different apoptosis regulators from both intrinsic and extrinsic pathways. A TaqMan-based custom array was designed consisting of several tumor suppressor genes and regulatory genes from various signal transduction pathways. PCR array was run on QuantStudio3 and analyzed by the ΔΔ*C*_T_ method using DataAssist™ software from Thermo Fisher. The data were normalized using 18s rRNA expression (apoptosis array) and global normalization (custom array). RQ indicates the fold change in gene expression against untreated control after normalization with the selected endogenous gene.

### Docking of anti-apoptotic proteins with quercetin

Anti-apoptotic proteins BCL2 (PDB ID:2o22), BCL-xl (PDB ID: 2YXJ) and MCL1 (PDB ID: 6O6F) with co-crystallized inhibitors were retrieved [32–34]. Quercetin was retrieved from zinc database in mol2 format. The protein chains were dockprepped and docked with quercetin using SwissDock docking server [35]. The docked poses where chosen on the basis of the least energy (lowest fullfitness) values and compared with the docked pose of the co-crystallized known inhibitor bound to the protein using the visualizing software, UCSF Chimera [36].

### Quantitative analysis of proteins involved in apoptosis and regulatory pathways using proteome profiler

The Human Apoptosis Array (R & D systems) was used to detect the expression of apoptosis-related proteins. Briefly, 1 × 10^7^ cells were plated and treated with 25 and 50 quercetin for 48 h. After treatment, cells were harvested, and lysates were extracted according to the manufacturer’s instructions. The total protein quantity in the lysates was estimated by Pierce BCA assay according to the manufacturer’s protocol. The amount of lysate equal to 400 μg of protein was used for the proteome array. Antibody-spotted nitrocellulose membrane was incubated overnight with the cell lysates and the array was processed according to manufacturer’s protocol to enable chemiluminescent detection of proteins. Likewise, the Human Oncogene Array was processed following the manufacturer’s protocol. The image of the blot was captured by chemiluminescent gel doc system (Bio-Rad, U.S.A.), and analyzed using ImageLab software. The intensity of the blot corresponds to the expression of the protein.

### Statistical analysis

All the data are expressed as means ± SD of at least three experiments. One-way ANOVA followed by *t* test was adopted for the statistical evaluation of the results. Significant differences were established at *P*≤0.05.

## Results

### Differential reduction in viability of Hela cells in a time- and dose-dependent manner

**Figure 1:**
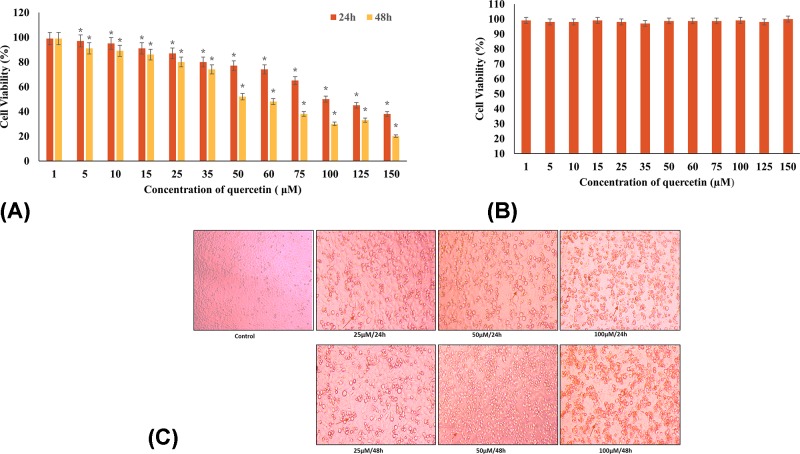
Quercetin induces differential cell viability (**A**) Cell viability assay using the MTT assay: dose- and time-dependent cytotoxicity of quercetin (1–150 µM) treatment on HeLa cells for 24 (red) and 48 h (blue). The EC_50_ of quercetin was found to be 100 µΜ at 24 h (*P≤0.05). (**B**) Cell viability assay: dose-dependent viability of quercetin (1–150 µM) treatment on lymphocytes. Quercetin was found to have no effect on cell viability. (**C**) Morphological changes in HeLa cells at varying concentrations of quercetin. Microscopic features of HeLa cells treated with different concentrations (25, 50 µM for 24 and 48 h) of quercetin (magnification 20×). Arrows indicate the rounding of cells (indicative of death) with increasing concentrations.

The effect of quercetin on the viability of HeLa cells was determined by treating the cells with different concentrations of quercetin and evaluating the cell viability using MTT assay. HeLa cells when treated with increasing concentrations of quercetin (1–150 µM) for 24 and 48 h showed significant growth inhibition in a dose- and time-dependent manner. The concentration at which quercetin inhibited the viability by 50% (EC_50_) was 100 µM after 24 h treatment (Figure 1A). The results indicate that quercetin has a significant inhibitory effect on the growth of HeLa cells in comparison with the untreated controls. Quercetin (25 µM) treatment reduced viability by 13 and 20% after 24 and 48 h, respectively. Whereas, 50 µM quercetin brought approximately 23% and 48% reduction in viability after 24 and 48 h exposure, respectively. The lymphocytes showed no adverse response against quercetin treatment (1–150 µM for 24 h) and did not inhibit their growth at the concentrations tested (Figure 1B). The results indicate that quercetin is safe on non-tumor cells and appears to specifically target cancerous cells.

The microscopic examination of the cells treated with various concentrations of quercetin for 24 and 48 h in comparison with untreated control showed the characteristic rounding off of the dying cells (Figure 1C).

### Quercetin restricts colony formation in HeLa cells

**Figure 2:**
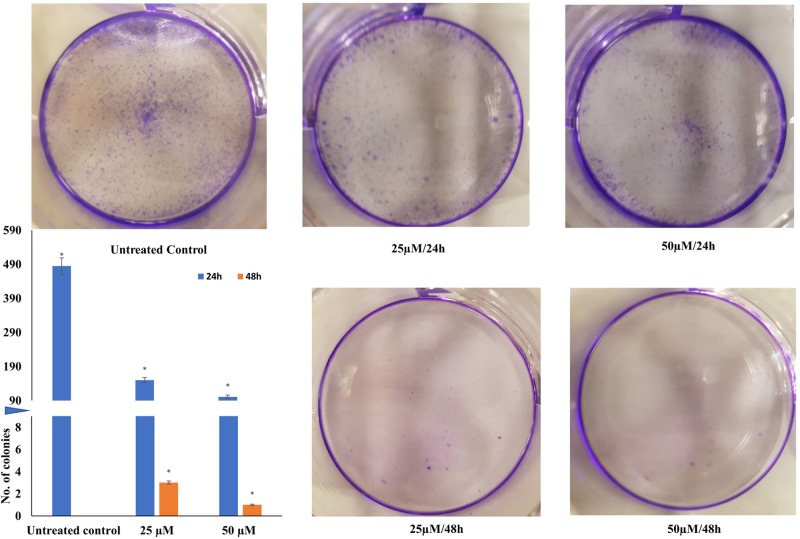
Colony formation assay: HeLa cells treated with different concentration (25, 50 µM for 24 and 48 h), counted (500 cells) and plated Colonies were monitored microscopically and photographed after 2 weeks. Colonies formed after 2 weeks were counted and the mean represented as a graph, which is inset. A split Y-axis graph has been used to clearly indicate all the values. Axis is split at value 8 and restarts at value 90 (*P≤0.05).

In order to examine the impact of quercetin on colony formation capacity of HeLa cells, 500 treated cells were seeded on to six-well plates. The untreated cells were used as control and to calculate the plating efficiency. While untreated cells formed colonies bearing approximately 50 cells each within 10 days, quercetin was observed to have impacted the ability of the cells to form colonies. The cells treated with 25 and 50 µM quercetin for 24 h formed 150 and 100 colonies, respectively. When treatment was extended to 48 h, no colonies were observed. The results indicate that quercetin is anti-proliferative and cytostatic (Figure 2).

### Quercetin induces nuclear morphology changes in HeLa cells

**Figure 3:**
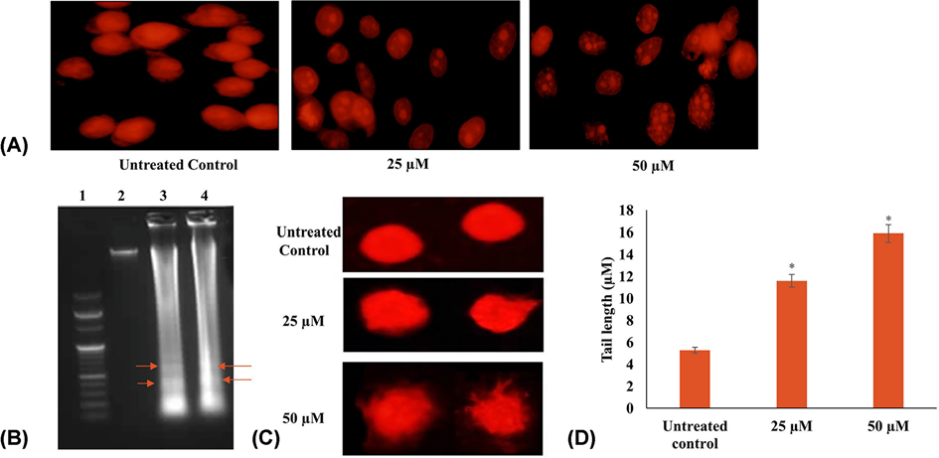
Quercetin induces apoptosis in HeLa cells (**A**) Nuclear morphological features of HeLa cells treated with different concentrations (25, 50 µM for 24 and 48 h) of quercetin (magnification 100×). Figures indicate nuclear condensation, fragmentation and formation of apoptotic bodies indicative of apoptosis. (**B**) DNA ladder assay: HeLa cells treated with different concentrations (25, 50 µM for 24 h) of quercetin were found to produce a DNA laddering pattern consistent with apoptosis. (**C**) Single cell gel electrophoresis assay: HeLa cells treated with different concentrations (25, 50 µM for 24 h) of quercetin induce DNA damage. (**D**) The tail length of the comets are represented as a graph and indicate extent of damage (*P≤0.05).

Following PI staining, fluorescent microscopy was used to observe apoptotic changes in more detail. Quercetin (25 and 50 µM treatment for 24 and 48 h) produced a substantial increase in nuclear condensation, nuclear fragmentation and apoptotic bodies in a dose- and time-dependent manner (Figure 3A).

### Quercetin induces DNA fragmentation in HeLa cells

Apoptotic cells demonstrate ladder formation due to the fragmentation of DNA. Quercetin (25 and 50 µM for 24 h) was found to cause DNA fragmentation in HeLa cells in comparison with untreated control confirming the induction of apoptosis (Figure 3B).

### Quercetin induces comet formation in HeLa cells

In order to understand the impact of quercetin on DNA damage, alkaline comet assay was performed on HeLa cells treated with 25 and 50 µM of quercetin for 24 h. The untreated cells yielded negligible DNA damage in the cells; whereas quercetin induced single- and double-stranded breaks in the DNA in a dose-dependent manner as observed in Figure 3C. The comet tail length was calculated and represented as a graph (Figure 3D).

### Quercetin induces cell cycle arrest in HeLa cells in a dose- and time-dependent manner

**Figure 4:**
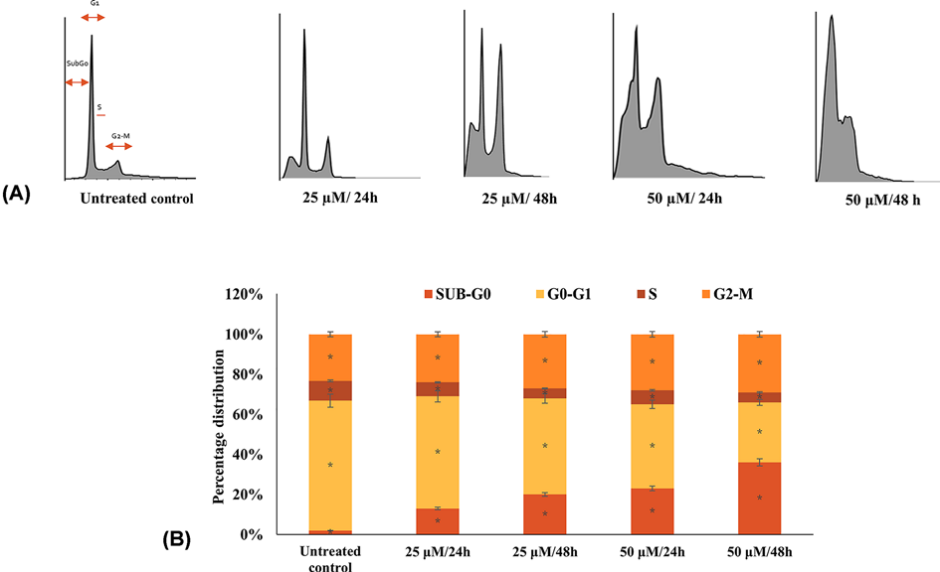
Flow cytometry: cell cycle of HeLa cells treated with different concentrations (25, 50 µM for 24 and 48 h) of quercetin was analyzed after staining with PI (**A**) Quercetin induces G_2_/M arrest with increase in sub-G_0_ apoptotic population. (**B**) The distribution of cells across the cell cycle is represented as a graph.

In order to understand the influence of quercetin on the cell cycle, HeLa cells were treated with quercetin (25 and 50 µM treatment for 24 and 48 h) and analyzed by flow cytometry. The results indicate that quercetin induces cell cycle arrest in G_2_-M phase with the G_2_-M population increasing proportional to the dose and duration of treatment (Figure 4). Further, the sub-G_0_ population, which is representative of apoptotic cells was also found to increase in a dose- and time-dependent manner (13% and 23% with 25 and 50 µM quercetin treatment for 24 h).

### Quercetin mitigates HeLa cell migration as evidenced in scratch wound assay

**Figure 5:**
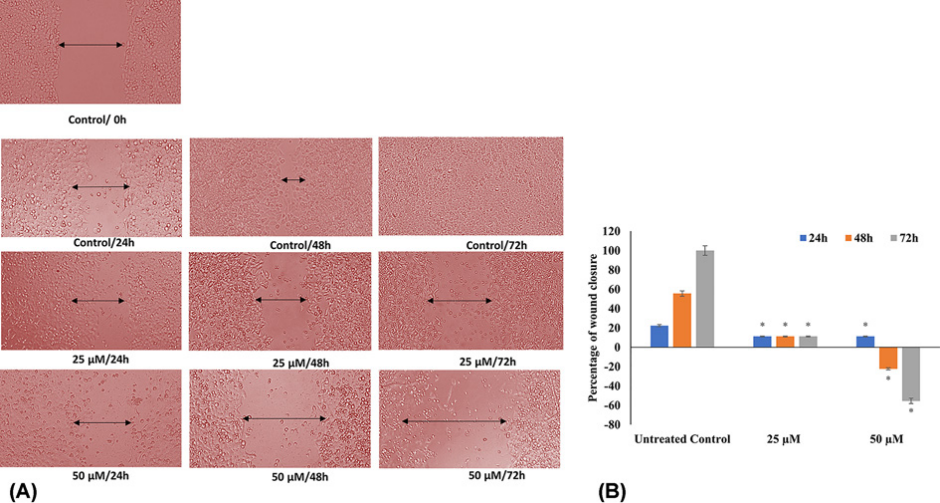
Scratch wound assay: HeLa cells treated with different concentrations (25, 50 µM) of quercetin and the migration of the cells across the cell free line was monitored microscopically (**A**) Images of the wound were obtained at 0, 24, 48 and 72 h. (**B**) The wound width was measured, and the percentage of wound closure was calculated and represented as a graph (*P≤0.05).

In the metastatic cascade, the migration of tumor cells is a significant occurrence. The scratch-wound assay demonstrated a significant reduction in the migration capacity of HeLa cells treated with quercetin compared with the controls (Figure 5). While, the untreated HeLa cells showed complete wound closure by 72 h; the cell-free line remained clear at concentrations above 25 µM. In 50 μM quercetin treated well, the acellular line was persistent, showing that there was no migration even after 72 h.

### Quercetin increases caspase 3 activity in a dose- and time-dependent manner

**Figure 6:**
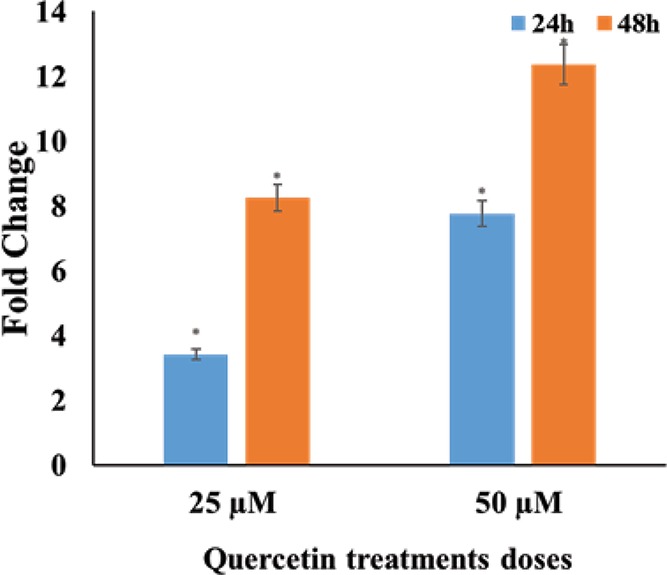
Caspase 3 activity: HeLa cells treated with different concentrations (25, 50 µM for 24 and 48 h) of quercetin increase the activity of caspase 3 The fold change with respect to untreated control is represented as a fold change (*P≤0.05).

In order to examine the ability of quercetin in affecting the activity of the central executioner caspase, caspase 3 was assessed by ELISA-based activity assay. Quercetin was found to increase the activity of caspase in a dose- and time-dependent manner. Fold change was calculated in comparison with the untreated controls. A significant fold change, 8- and 12-folds was observed against 25 and 50 µM quercetin treatment of 48 h, respectively (Figure 6).

### Quercetin induces apoptosis in HeLa cells mainly via extrinsic pathway

In order to ascertain the mechanism by which quercetin induces apoptosis, the expression of various genes involved in apoptosis was studied by TaqMan-based Real time qPCR array. Genes having RQ greater than 1.5 were considered as up-regulated; and those with RQ lower than 0.5 were considered as down-regulated.

**Figure 7:**
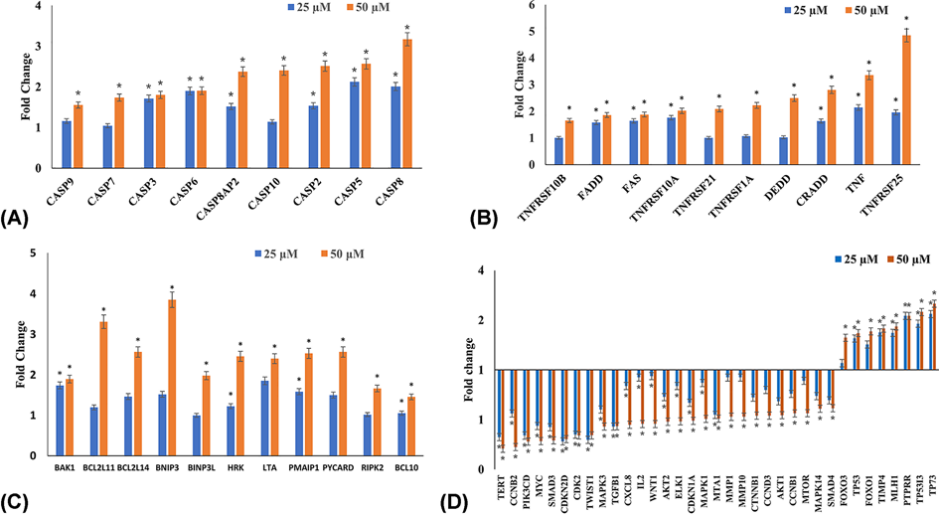
Expression analysis of genes involved in apoptosis and cell signaling after treatment with 25, 50 µM of quercetin for 48 h (**A**) RQ plot of caspases. (**B**) RQ plot of extrinsic receptors and ligands. (**C**) RQ plot of pro-apoptotic genes. (**D**) RQ plot of cell cycle regulators, tumor suppressors and genes involved in PI3K, MAPK and WNT signaling (*P≤0.05).

The results (Figure 7) indicate that the genes involved in the extrinsic pathway of apoptosis are up-regulated and therefore this could be the mechanism through which quercetin induces apoptosis. The expression of TRAIL, FASL, TNF and their receptors (FAS, TNFSF10, TNFRSF10A, TNFRSF10B, TNFRSF1A, TNFRSF1B, TNFRSF21, TNFRSF25) increased and mediated the extrinsic pathway. TRADD, CRADD, DEDD were also found to be elevated. Further increase in expression of caspase 8, 10, 3 and 7 indicate the role of the extrinsic pathway. Caspase 8 and 10 expression via the normal course of the extrinsic pathway should lead to the activation of caspase 3 and 7, which are the effector caspases. The expression of caspase 9, 2, 14, and 6 were also found to be increased. Additionally, several genes from BCL2 family, which are involved in the pro-apoptotic action were also found to be elevated such as BAK1, HRK, NOXA, BIM, BCL10 and BCL2L14. Other pro-apoptotic responders such as BNIP3, BNIP3L, LTA, PYCARD and RIPK2 were found to increase in response to quercetin. The expression of several tumor suppressor genes involved in anti-proliferative and apoptotic response such as FOXO1, FOXO3, TP53, TP5313, TP73 were found to be increased.

### Quercetin modulates expression of genes involved in cell cycle regulation

Quercetin down-regulates genes involved in G_2_-M stage of the cell cycle, viz. CCNB1, CCNB2 and CDK2, which is consistent with the observed G_2_-M arrest. Further it down-regulates CCND3 and CDKN1A but does not impact other genes involved in G_1_ stage. Telomerase reverse transcriptase (TERT) expression was also significantly down-regulated (Figure 7D).

### Quercetin modulates expression of genes involved in migration

Quercetin down-regulates genes involved in migration and invasion, viz. MMP14, MMP9 and MTA1; while up-regulating TIMP4. TWIST1, an inhibitor of e-cadherin, was also steeply down-regulated (Figure 7D).

### Quercetin suppresses MAPK, PI3K and WNT pathways to bring out its anti-proliferative and anti-migratory effect

ELK1 and MEKK/MAP3K5 gene expressions are down-regulated along with an increase in PTPRR, which is an inhibitor of MAP pathway. A significant decrease in AKT1, AKT2, MTOR, PI3KCTB and PI3KCD was observed, which suggests an inhibition of the PI3K pathway. WNT pathway is also suppressed by quercetin as evidenced by the decreased expression of CTNNB1 and WNT2. Gene expression of SMAD2, SMAD3, SMAD4 and TGFβ1, which play significant roles in WNT pathway, are also down-regulated. An increase in VHL expression was also found. It further down-regulates the genes involved in inflammation such as CXCL8, MYC, IL2 and IL1A (Figure 7D).

### Quercetin modulates expression of pro- and anti-apoptotic proteins

**Figure 8:**
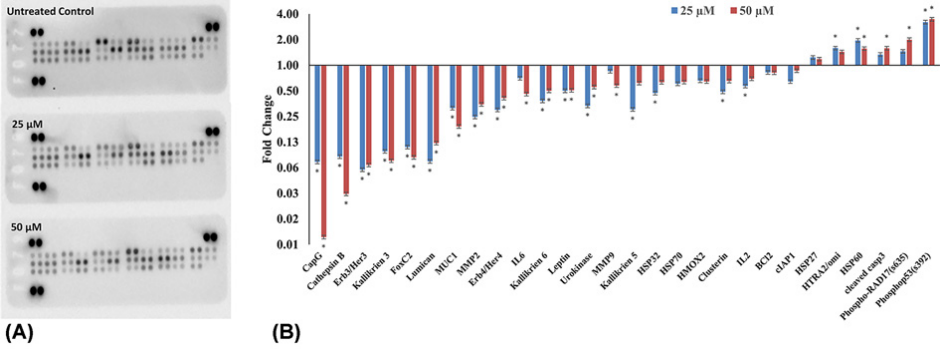
Quercetin induces apoptosis in HeLa cells Proteome profiler: (**A**) image of the proteome profiler membrane showing differential protein expression. (**B**) Proteins involved in apoptosis and regulatory pathways were quantitated after treating HeLa cells with 25, 50 µM of quercetin for 48 h and represented as fold change over control. Quercetin increased pro-apoptotic proteins and decreased anti-apoptotic proteins (*P≤0.05).

Proteome profiler-based quantitation of the proteins involved in apoptosis, cell cycle regulation showed modulation consistent with mRNA expression. Cleaved caspase3, FAS, HTRA2/omi, phospho53(s392), phospho-RAD17(s635) and endolglin are up-regulated, whereas cIAP1, clusterin, HSP32, HMOX2, HSP70, CapG, cathepsin B, Erb3/Her3, Erb4/Her4, FoxC2, IL2, IL6, kallikrien 3, kallikrien 5, kallikrien 6, leptin, lumican, MMP2, MMP9, MUC1 and urokinase are down-regulated. The fold changes with respect to untreated control are plotted in Figure 8.

### Quercetin modulates anti-apoptotic proteins

**Figure 9:**
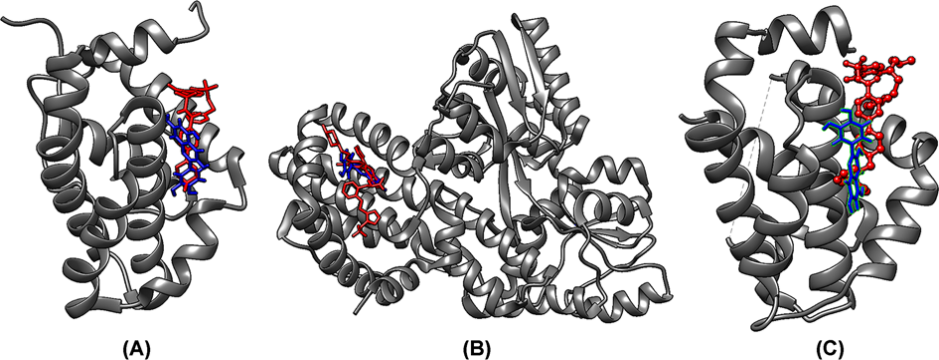
Docking analysis of anti-apoptotic proteins with quercetin (blue) co-crystallized inhibitor (red) shows that quercetin occupies the same region as the inhibitor and could inhibit the proteins (**A**) BCL2; (**B**) BCLxl; (**C**) MCL1.

Quercetin was docked with BCL2, BCL-xl and MCL1 proteins. The docked poses were selected on the basis of least energy (lowest fullfitness) values. The docked pose of quercetin was compared with the co-crystallized known inhibitor bound to the protein (Figure 9). The interaction between quercetin and protein in each case was similar to that of the established inhibitor in the X-ray crystal structure; with high degree of shared amino acid residues. The region in which the co-crystallized inhibitor and quercetin are bound is responsible for interacting with the pro-apoptotic BCL2 proteins. These anti-apoptotic proteins bind to and sequester the pro-apoptotic proteins thereby limiting their activation [32,33,37]. Quercetin engages with these anti-apoptotic proteins in the same manner as the known inhibitors and could prevent their sequestering of the pro-apoptotic proteins. This will aid in apoptotic response.

## Discussion

Worldwide, the prevalence and mortality from cancer has been growing, even though our knowledge and treatment of diseases has progressed tremendously. Therefore, chemoprevention using natural dietary agents presents itself as a rational and appealing strategy. In the present study, we comprehensively analyze the anti-proliferative, pro-apoptotic and anti-migratory effect of quercetin on HeLa cells by modulating various signaling pathways.

Deregulation of the cell cycle with rampant proliferation while evading apoptosis and promoting metastasis are characteristics of cancer cells. An effective drug candidate should be able to limit cell proliferation, induce apoptosis and restrict migration. In the present study, the ability of quercetin to induce cytotoxicity in HeLa cells was established through the MTT assay and its EC_50_ was found to be 100 µM in 24 h. Whereas, other research groups have reported 50% cell viability in HeLa cells at 110.38 μM of quercetin in 18 h and 80 μM at 24 h [38,39]. Further, anti-proliferative and cytostatic ability of quercetin was demonstrated by a dose- and time-dependent inhibition of colony formation on quercetin treated HeLa cells (Figure 2). In comparison with the control it was found that quercetin limits colony formation after 24 h and almost no colonies were formed after 48 h of treatment. Earlier studies in different cell lines also showed similar findings [40–43]. Morphological analysis of quercetin treated HeLa cells by light microscopy and fluorescent microscopy (after PI staining) (Figures 1C and 3A) showed the characteristic changes associated with apoptosis including shrinkage, nuclear fragmentation, rounding off of dying cells and apoptotic bodies. To further assess the anti-proliferative and apoptosis-inducing effect of quercetin on Hela cells, flow cytometry and DNA fragmentation assay was performed. In our study, quercetin was found to induce G_2_-M arrest with accumulation of sub-G_0_ apoptotic population which is in line with previous findings of cell cycle arrest at G_2_-M in HeLa, breast carcinoma, leukemia and esophageal adenocarcinoma cell lines [39,44–46]. In leukemic cell line, NALM6 while sub-G_0_ accumulation of apoptotic population was found with increasing doses of quercetin, at lower concentrations, S-phase arrest was observed [47]. This shows that in some cell types, a variable response is observed. Our flow cytometry results are consistent with the observed morphological changes. Apoptotic induction was further supported by the DNA fragmentation assay. A clear DNA laddering pattern with bands was observed in quercetin treated HeLa cells in sharp contrast with the untreated control. Apoptosis can be initiated by the onset of DNA damage; in order to ascertain whether DNA damage could be partly responsible for the observed apoptotic response, Comet assay was performed. Mild DNA damage was mediated by quercetin in a dose- and time-dependent manner with marked increase in comet tail length by 50 µM quercetin in 48 h. Earlier studies also support both these observations [43,48,49].

Apoptosis can be induced by one of two core pathways, the extrinsic and the intrinsic pathways, with both requiring the activation of caspase proteins [50–53]. Quercetin increases the transcription of the extrinsic pathway death receptors and ligands including TNF, DR4, DR5, FAS as well as FASL and TRAIL. TRAIL and TNF receptors are important starting points for extrinsic apoptosis [51]. The initiator caspases consisting of caspase 2, -8, -9 and -10 were up-regulated by quercetin in a dose-dependent manner with caspase 8 reaching an RQ of 3.6 when treated with 50 μM quercetin for 48 h. The function of caspase 8 is an important step in TNF-induced extrinsic apoptotic pathway, which was also found to be up-regulated. The up-regulation of the receptors and caspases indicated that quercetin induces the extrinsic pathway of apoptosis. The intrinsic pathway involves the release of cytochrome *c* from the mitochondria, activation of caspase 9, eventually activating caspase 3 and other pro-apoptotic molecules [54,55]. Quercetin increases caspase 9 gene expression and cytochrome *c* protein levels marginally; indicating perhaps a lower emphasis on mitochondrial pathway. The effector caspases (caspase 3, 6 and 7) which produce the apoptotic indicators are up-regulated by quercetin in a dose-dependent manner. The increase in the levels of initiator and executioner caspases highlight the concordance in the apoptotic response. Of all the effector caspases, caspase 3 has a central role and is important for both PARP cleavage and DNA fragmentation [55]. It is significant therefore that quercetin increases both the transcription of caspase 3, and the functional cleaved caspase 3 protein in a dose-responsive manner (Figures 7 and 8). Further, the activity of caspase 3 in quercetin treated HeLa cells showed a steep increase, reiterating the evident activation of the caspase cascade and onset of apoptosis (Figure 6). Caspase 2 is required for DNA damage and is a substrate for both caspase 3 and caspase 8 [55]. The up-regulation of caspase 2 by quercetin is significant in that it further reiterates the functional activation of caspase 3 and caspase 8, as well as substantiates the results of the DNA fragmentation and comet assay.

The caspases are supported by the increased transcription of several genes involved in the apoptotic response (Figure 7). Quercetin was found to up-regulate the pro-apoptotic members, Bax, Hrk, Noxa, Bim, BCL10 and BCL2L14. It also mediates an increase in several pro-apoptotic proteins (Figures 7 and 8) including HTRA2/omi and endolglin. HTRA2/omi functions as an antagonist to inhibitors of apoptosis (IAPs) and aids in apoptosis [56]. It is further significant that quercetin increases s635 phosphorylated Rad17 (s635). The ability of Rad17 to trigger G_2_-M arrest and DNA damage induced apoptosis is dependent on s635 phosphorylation [57]. This further strengthens the earlier observations. Quercetin also up-regulates transcription of p53, p73 and p5313 and protein levels of phospho53(s392). P53 and its homolog p73 are silenced by HPV-E6 and play an important role in cell cycle and apoptosis [58–60]. Anti-apoptotic proteins such as cIAP1, Clusterin, HSP32, HMOX2, HSP70, CapG, Cathepsin B are down-regulated. Further, molecular docking experiments suggest that anti-apoptotic BCL2 family proteins (BCL2, BCL-xl and MCL1) could be directly inhibited by quercetin (Figure 9 and Table 2). A recent study showed that a quercetin–alanine conjugate directly binds to BCL2 and enhances apoptosis [61]. We believe that a similar interaction may be at play here.

This lends further support to the apoptotic outcome mediated by quercetin. In the present study, while both intrinsic and extrinsic pathways are activated by quercetin, a comparatively higher folds increase in caspase 8 and other proteins of the extrinsic pathway allow us to conclude that the extrinsic pathway could have the lead role with the mitochondrial pathway playing a supportive role. In leukemia cells (NALM6), quercetin was found to bring about mitochondrial pathway of apoptosis by increasing cytochrome *c*, caspase 9 and depolarization of mitochondrial membrane potential [47]. In breast cancer cells, quercetin induced caspase-dependent extrinsic apoptosis by up-regulating the levels of cleaved caspase-8 and caspase-3 without altering the mitochondrial membrane potential [62].

TERT, overexpressed in cervical cancer cells, determines telomere length and facilitates cancer cells to evade apoptosis and continue proliferation [63–65]. As further evidence to explain the anti-proliferative and cell cycle arrest mediated by quercetin, it was observed that the tested doses of quercetin promote a significant down-regulation in TERT transcript expression as well as down-regulates CCNB1, CCNB2 and CDK2 which are involved in cell cycle regulation.

Another hallmark of cancer, invasion and metastasis is responsible for most cancer-related mortality and morbidity and is thus an important therapeutic target. Quercetin’s ability to inhibit the migration is evidenced by the results of the scratch wound assay (Figure 5). At higher treatment dosages and durations, a cell-free line was maintained. This outcome is explained by the observed down-regulation of MMP14, MMP9, MTA1 and TWIST1 with simultaneous up-regulation of CDH1, TIMP3 and TIMP4. CDH1 is important for cell adhesion and when usually silenced by methylation can lead to metastasis and tumor progression [66–68]. Several studies have documented that increase in CDH1 can inhibit metastasis and cell growth [69,70]. TWIST1 acts as an inhibitor of CDH1 and is seen to be steeply reduced. MMPs promote tumor invasion and metastasis, while TIMPs oppose this action [71]. Thus, the concordant modulation of these genes by quercetin highlights the thoroughness of its anti-migratory effect.

The dysregulation of several signaling pathways such as the PI3K, WNT, MAPK, JAK/STAT help in cancer progression by promoting proliferation through growth stimulating signals, suppressing growth inhibitors, evading apoptosis and promoting metastasis. Quercetin was found to modulate the expression of several genes involved in these pathways; effectively causing inhibition of proliferation, migration and apoptosis (Figure 7 and Table 1). In the PI3K pathway, quercetin brings about decrease in transcript expression of AKT1, AKT2, MTOR, PI3KCTB and PI3KCD. AKT is an important molecule that can further activate other pathways including mTORC1 [72]. AKT is activated by several growth factors and cytokines through the receptor tyrosine kinases like HER by binding their cognate receptor tyrosine kinase and promotes cell survival by inactivating pro-apoptotic proteins and the forkhead (FoxO1/3a) transcription factors [73]. Interestingly, quercetin down-regulates the protein levels of HER3 and HER4 and a marked increase in the levels of the FOXO1/3 transcription factors. FOXO1/3 are tumor suppressors that induce the transcription of pro-apoptotic genes and death receptors involved in apoptosis, cell cycle regulation and DNA damage repair [74]. FOXO1 expression inhibits cervical cancer development by bringing about cell cycle arrest and apoptosis and is a favorable prognostic factor [75].

**Table 1:** Table of the genes modulated by quercetin to bring about its anticancer effect

Effect	Molecular target	Up-regulation	Down-regulation
Apoptosis	Caspases	CASP9, CASP7, CASP3, CASP6, CASP14, CASP8AP2, CASP10, CASP2, CASP5, CASP8	
	Pro-apoptotic BCL2 family	BAK1, HRK, PMAIP1, BCL2L14, BCL2L10, BCL2L11	
	Death receptors and ligands	TNFRSF10B, FADD, FAS, TNFRSF10A, TNFRSF21, TNFRSF1A, DEDD, CRADD, TNF, TNFRSF25, FASLG, TNFSF10, TRADD	
	Other pro-apoptotic proteins	BNIP3, BNIP3L, LTA, PYCARD, RIPK2	
	Signaling pathway and TSG	FOXO3, TP53, FOXO1, TIMP4, MLH1, PTPRR, TP53I3, TP73, CDH1, SOCS1	TERT, CCNB2, PIK3CD, MYC, SMAD3, CDKN2D, CDK2, TWIST1, MAPK3, TGFB1, CXCL8, IL2, WNT1, AKT2, ELK1, CDKN1A, MAPK1, MTA1, MMP1, MMP10, CTNNB1, CCND3, AKT1, CCNB1, MTOR, MAPK14, SMAD4
	Protein expression	Cleaved CASPASE3, FAS, HTRA2/OMI, phospho53 (S392), phospho-RAD17 (S635), Endolglin	CIAP1, CLUSTERIN, HSP32, HMOX2, HSP70, CAPG, CATHEPSIN B, ERB3/HER3, ERB4/HER4, FOXC2, IL2, IL6, KALLIKRIEN 3, KALLIKRIEN 5, KALLIKRIEN 6, LEPTIN, LUMICAN, MMP2, MMP9, MUC1, UROKINASE
Cell cycle regulation and anti-proliferation	Cell cycle regulatory genes		CCNB2, CDKN2D, CDK2, CDKN1A, CCND3
	Anti-proliferation genes		TERT
Anti-migration	Anti-metastatic genes	CDH1, TIMP4, SOCS1	MMP1, MMP10
Anti-proliferation,			
anti-metastatic	PI3K pathway		AKT2, AKT1, MTOR
	WNT pathway		CTNNB1, TGFB1, WNT1, SMAD4
	MAPK pathway	PTPRR	MAPK3, MAPK1, MAPK14, ELK1
Anti-inflammation	Inflammation markers		CXCL8, IL2, MYC

**Table 2:** Interaction of least energy docked pose of quercetin with anti-apoptotic proteins

Protein (PDB ID)	Full fitness value	Interacting residues within 5A of quercetin	Predicted pattern of interaction
BCL2, (PDB ID 2O22)	−1446.2208	PHE 101, TYR 105, ASP 108, PHE 109, MET 112, LEU 134, ASN 140, GLY 142, ARG 143, ALA 146, PHE 150	Inhibitory. Similar to co-crystallized inhibitor
BCL xl, (PDB ID 1R2D	−869.9743	PHE 97, TYR 101, ARG 103, PHE 105, ASP 107, LEU 108, GLN 111, GLU 129, LEU 130, PHE 131, ARG 132, ASP 133, GLY 134, ARG 139, ALA 142	Inhibitory. Similar to co-crystallized inhibitor
MCL1, (PDB ID 5LOF)	−2807.5872	HIS 224, ALA 227, PHE 228, MET 231, MET 250, VAL 253, PHE 254, ARG 263, ILE 264, THR 266, LEU 267, PHE 270	Inhibitory. Similar to co-crystallized inhibitor

WNT signaling pathway plays an important role in cervical cancer and regulates tumor progression, particularly migration [76]. WNT2 expression is up-regulated in cervical cancer and is associated with tumor size, cell motility and invasion [77]. Quercetin reduces expression of WNT2 and CTNNB1, which are important moieties in the WNT pathway. TGFβ1, SMAD2, SMAD3 and SMAD4 are also down-regulated by quercetin. TGFβ/SMAD signaling is linked to EMT, migration and invasion [78]. The TWIST gene, part of the WNT pathway inhibits CDH1 [79,80]. As noted earlier, quercetin decreases TWIST1 and increases CDH1.

MAPK pathway is centrally involved in cell proliferation; while several members showed a decreasing trend, significant gene expression reduction was observed with ELK1 and MEKK/MAP3K5, alongside increase in PTPRR, which is an inhibitor of MAP pathway. Cervical cancer cells carry aberrantly high methylation rates of PTPRR [81]. ELK1 is involved in up-regulating the oncogene, c-fos and activating the cell cycle [82]. Therefore, its notable that these are down-regulated by quercetin.

Inflammation and inflammatory responses are negative regulators of cancer therapeutics and it is pertinent that the tested dosages of quercetin down-regulate the expression of tumor markers and proteins involved in inflammation. Quercetin reduces the expression of CXCL8, IL2, IL8 and IL6. CXCL8, a proinflammatory oncogene is highly expressed in cervical cancer tissues [83]. IL-2, IL-8 and IL-6 expression is significantly correlated with poor prognosis [84–86]. Caspase 1 and 4 are involved in inflammatory response and is not central to the apoptotic response [87]. In this regard, it is interesting that quercetin does not change the expression of caspase 1 as well as 4.

Additionally, we found that quercetin is differentially cytotoxic and does not affect the viability of lymphocytes (Figure 1B). The safe profile of quercetin in normal cell lines and animal models has been validated in other studies as well [47,48,88,89]. The specificity of quercetin’s cytotoxic action against tumor cells while not impacting normal cells, makes it an ideal drug candidate.

## Conclusion

The findings of the present study show that quercetin systematically alters the PI3K, MAPK and WNT pathways by modulating the expression of several proteins leading to the inhibition of cell proliferation, cell cycle arrest, DNA damage and apoptosis in cervical cancer (HeLa) cells. A promising alternate route to cancer chemoprevention and treatment strategies appears to be the use of dietary polyphenols such as, quercetin. The present study provides emphatic evidence for the potential use of quercetin as a multipronged anticancer therapeutic agent.

## Abbreviations

EMT: epithelial to mesenchymal transition
FASL: Fas ligand
PARP: Poly (ADP ribose) polymerase 1
PI: propidium iodide
PI3KCD: phosphotidyl-inositol-4,5-bisphosphate 3-kinase catalytic subunit delta
PTPRR: protein tyrosine phosphatase receptor type R
qPCR: quantitative real time polymerase chain reaction
TERT: telomerase reverse transcriptase
TIMP: tissue inhibitors of metalloproteinases
Wnt: Wingless
